# Using a DAS28-CRP-steered treat-to-target strategy does not eliminate subclinical inflammation as assessed by ultrasonography in rheumatoid arthritis patients in longstanding clinical remission

**DOI:** 10.1186/s13075-021-02426-w

**Published:** 2021-02-01

**Authors:** Lene Terslev, Cecilie Heegaard Brahe, Mikkel Østergaard, Viktoria Fana, Mads Ammitzbøll-Danielsen, Torsten Møller, Simon Krabbe, Merete Lund Hetland, Uffe Møller Døhn

**Affiliations:** 1 Copenhagen Center for Arthritis Research (COPECARE), Center for Rheumatology and Spine Diseases, Copenhagen University Hospital, Rigshospitalet, Glostrup, Copenhagen, Denmark; 2 Department of Clinical Medicine, University of Copenhagen, Copenhagen, Denmark

**Keywords:** Remission, Ultrasound, Doppler, Treat-to-target, Subclinical synovitis

## Abstract

**Background:**

Subclinical synovitis by ultrasound is a frequent finding in rheumatoid arthritis (RA) patients in remission and has been shown to be related to erosive progression, risk of flare and unsuccessful drug tapering, but it has not been investigated how a DAS28 T2T-steered strategy in routine care affects the presence of subclinical synovitis in RA patients in remission. The aim of the current study was to investigate the presence of ultrasound-detected subclinical inflammation in RA patients in long-term remission receiving either biological or conventional disease-modifying anti-rheumatic drugs (bDMARD/csDMARD) and, finally, to investigate the presence of ultrasound remission using different ultrasound remission criteria.

**Methods:**

Eighty-seven RA patients (42 patients receiving bDMARD and 45 csDMARD) received DAS28-CRP-steered treatment in routine care and had achieved DAS28-CRP-remission for > 1 year without radiographic progression. Twenty-four joints were scored 0–3 by ultrasound (elbows, wrists, knees, ankles, metacarpophalangeal and metatarsophalangeal joints 2–5) for grey-scale synovial hypertrophy (GS) and colour Doppler activity (CD) using the OMERACT scoring system. Ultrasound remission was defined as strict (GS score = 0 and CD score = 0), semi-strict (GS score < 1 and Doppler score = 0) and Doppler remission (Doppler score = 0).

**Results:**

No differences between treatment groups were found for GS sum score and Doppler sum score (median (range) 6 (0–19) and 0 (0–12), respectively). A Doppler score > 0 in at least 1 joint was seen in 44%, a GS score > 1 in at least 1 joint in 93% and a GS score > 2 in at least 1 joint in 54% of patients. Strict ultrasound remission was only observed in bDMARD patients (7%; *p* = 0.01). Thirty-seven per cent were in semi-strict ultrasound remission and 56% in Doppler remission (no significant difference between groups) with similar results across the subgroups of patients who also fulfilled the ACR-EULAR Boolean-, CDAI- and SDAI-remission criteria.

**Conclusions:**

Ultrasound frequently detected subclinical synovitis in RA patients in longstanding DAS28-remission obtained through a DAS28-CRP-steered strategy. This was independent of treatment and applied ultrasound remission criteria. Strict ultrasound remission was rare.

## Background

The treatment goal in patients with rheumatoid arthritis (RA) is to rapidly suppress inflammation and thereby preventing pain and joint destruction and improving functional ability and quality of life. This was addressed in the EULAR 2010 treat-to-target (T2T) recommendations, where the treatment target was specified as clinical remission or at least low disease activity, assessed by the use of composite measures and when not achieved, adjustment of therapy is recommended [1]. Targeting remission by applying a disease activity score based on a 28-joint count (DAS28) < 2.6 as compared to a conventional strategy not using a DAS28-targeted strategy has shown a significant advantage favouring the T2T DAS28 approach [2, 3].

The updated EULAR 2016 T2T recommendations favour ACR-EULAR Boolean-remission over DAS28-remission [4], because it is more stringent and better reflects the clinical perception of remission, i.e. the absence of signs and symptoms of significant inflammatory disease activity. DAS28-CRP is still, however, applied in routine care and clinical trials [5]. Despite data showing that subclinical inflammation as detected by ultrasound and magnetic resonance imaging is present in a substantial proportion of patients with RA in clinical remission [6–12], it was decided not to include imaging remission into the updated EULAR T2T recommendations, leaving clinical remission as the therapeutic target [4]. However, data shows that subclinical synovitis detected by ultrasound seems to be independent of the applied clinical remission criteria and type of treatment (conventional synthetic disease-modifying anti-rheumatic drugs (csDMARD) or biological DMARD (bDMARD)) [12–16].

Subclinical synovitis has been shown to be related to erosive progression as well as to the risk of flare and unsuccessful drug tapering, especially when Doppler activity is present [7, 8, 17–22]. Ultrasound remission is therefore generally perceived as an ultrasound disease state without Doppler activity, but different studies put different emphasis on the presence of synovial hypertrophy by grey-scale (GS) ultrasound. Though subclinical synovitis in patients in remission has been assessed previously, none of the studies evaluated patients in longstanding remission obtained through a DAS28 T2T-steered strategy applied in routine care [6, 7, 12–16].

Therefore, the aims of this study were, in RA patients in longstanding remission obtained by a DAS28CRP T2T-steered strategy in routine care, to investigate if the presence of ultrasound detected subclinical inflammation was less than previously reported; secondly, in this T2T-steered cohort, to assess if bDMARD-treated patients had less subclinical synovitis than csDMARD-treated patients, using both a comprehensive joint assessment and an assessment of the hands only; finally, to investigate the proportion of patients in ultrasound remission when applying three different ultrasound remission criteria.

## Material and methods

The study is a cross-sectional, observational study in patients with RA in sustained DAS28CRP-remission treated with either csDMARD only or bDMARD treatment (as monotherapy or in combination with csDMARD). Patients were recruited from the Center for Rheumatology and Spine Diseases at Rigshospitalet, Glostrup, Denmark. Clinical evaluation and ultrasound examination were conducted on the same day, at the time of inclusion. The local ethics committee found that a formal ethical approval was not needed because of the observational design (H-2-2014-FSP53). Despite this, all patients gave written informed consent and the study was conducted in accordance with the Declaration of Helsinki.

The inclusion criteria were RA fulfilling the 2010 EULAR/ACR classification criteria with DAS28-CRP < 2.6 for at least 1 year documented in at least 3 clinical visits in the DANBIO registry, age > 18 years, stable treatment with no indication for treatment change for at least 1 year and no glucocorticoid treatment 6 months prior to inclusion and no radiographic progression during the previous year assessed with the x-ray of hands and feet by the radiology department.

### Clinical evaluation

The national DANBIO registry is an integrated part of the routine clinical monitoring of RA patients in Denmark [23, 24]. The DANBIO registry is used as a clinical tool in all departments to monitor arthritis patients and their response to treatment or lack thereof and notifies when remission is not reached. For each clinical visit, swollen and tender joint counts of at least the 28 joints included in the DAS28CRP (bilateral wrist, metacarpophalangeal joints (MCPs), proximal interphalangeal joints (PIPs), elbow, shoulder and knee joints) are entered into DANBIO together with visual analogue scale (VAS, 0–100 mm) assessments of pain and patient’s and physician’s global assessment, Health Assessment Questionnaire (HAQ) and C-reactive protein (CRP). From the results of physical examination, patient questionnaires and CRP, the DAS28CRP and the CDAI are automatically calculated at each clinical visit informing the clinician whether DAS28 and/or CDAI-remission is obtained. If DAS28CRP > 2.6, there is an automatic alert to the clinician to change/optimise treatment until DAS28CRP < 2.6 has been reached. Patients are seen in the out-patient clinic approximately every 3 months to ensure treatment optimisation until remission is obtained. Treatment is escalated according to national T2T recommendations in routine care.

### Ultrasound

A GE Logiq® E9 R5 (Milwaukee, WI, USA) ultrasound machine with a 5-16ML linear array transducer was used for all examinations. Colour Doppler settings for slow flow [25] were kept unchanged throughout the study. The Doppler frequency was set at 8.2 MHz, PRF at 0.4 kHz, wall filter 57, colour priority at 100% and Doppler gain just below the noise level. The size and position of the colour box was set to go to the top of the image to recognise artefacts caused by vessels above the joint.

#### Ultrasound examination

The ultrasound examinations were performed by experienced ultrasonographers (LT, VF, TM, UMD and MA) with more than 7 years of experience in musculoskeletal ultrasound and scoring of synovitis in clinical and research settings. Prior to the study, reader exercises on scoring joint inflammation were conducted in static images to ensure agreement between observers.

Ultrasound examination techniques were in line with the EULAR scanning guidelines with regard to patient position and scanning planes [26]. In total, 24 joints were evaluated (bilateral elbows, wrists (radio-carpal, inter-carpal and the radioulnar joint—using the highest score of the three), MCP 2–5, knees, talo-crural joints and metatarsophalangeal joints (MTPs) 2–5).

The examination time was approximately 20 min per patient and representative images of all pathology were stored for each patient. The clinical examination was performed prior to the ultrasound examination by a rheumatologist different to the sonographer who was kept blinded to the results of the clinical examination and laboratory findings.

#### Image evaluation

GS and colour Doppler (CD) ultrasound were used to assess synovial hypertrophy and synovial vascularization using the OMERACT definition for synovitis [27] and the EULAR-OMERACT semi-quantitative scoring system (0–3) for grading the two synovitis components (GS and CD) [27, 28]. A score was noted per joint for each modality in order to assess different severities of synovial hypertrophy and CD activity. In addition, a total grey-scale sum score and a Doppler sum score were calculated for each patient (sum of the scores for all joints with a maximum score of 72). Ultrasound synovial hypertrophy was defined as a GS score > 1 and synovial hypervascularization as a Doppler score > 1.

#### Defining ultrasound remission

Ultrasound remission status was assessed using three different definitions: *strict ultrasound remission* (GS score = 0 and Doppler score = 0), *semi-strict ultrasound remission* (GS score < 1 and Doppler score = 0) and *Doppler ultrasound remission* (Doppler score = 0, irrespective of the GS score).

### Statistics

Demographic data are presented as median and range. Comparisons between groups were done using Mann-Whitney’s test for continuous data and Pearson chi-square for categorical data with a *p* value < 0.05 considered statistically significant. SPSS version 25 was used for statistical analysis.

## Results

Eighty-seven consecutive patients with RA in DAS28-CRP-remission for at least 1 year were included; 45 patients received csDMARDs (csDMARD group) while 42 patients received bDMARD either alone or in combination with csDMARDs (bDMARD group). Demographic data for the entire cohort stratified by type of treatment may be seen in Table 1. For all patients, the median DAS28CRP was 1.8 (range 1.1–2.5) and significantly lower in the csDMARD-treated group compared to the bDMARD-treated group. Overall, in the bDMARD group, eight patients (19%) received bDMARD monotherapy, while the rest received combination therapy with one csDMARD. In the entire cohort, 65 (75%) patients also fulfilled SDAI-remission criteria (SDAI ≤ 3.3), and 67 (77%) CDAI-remission criteria (CDAI ≤ 2.8). All patients fulfilling the CDAI-remission criteria also fulfilled the ACR-EULAR Boolean-remission criteria and vice versa.

**Table 1:** Clinical and ultrasound data for the patients in DAS28CRP-remission > 1 year obtained through a treat-to-target steered strategy

	All patients (***n*** = 87)	csDMARD group (***n*** = 45)	bDMARD group (***n*** = 42)	Difference between groups (***p*** values)
Age (years)	61 (25–82)	64 (31–82)	57 (25–82)	0.14
Female gender	66%	62%	69%	0.50
Disease duration (years)	10 (1–54)	6 (1–44)	13 (0–2)	< 0.001
Positive rheumatoid factor	61%	56%	67%	0.29
Positive anti-CCP	67%	56%	79%	0.02
Erosive disease	66%	51%	81%	0.003
Number of current DMARDs	1 (0–3)	1 (0–3)	1 (0–1)	0.003
**Clinical assessment**				
Swollen joint count (0–28)	0 (0–2)	0 (0–2)	0 (0–2)	0.71
Tender joint count (0–28)	0 (0–1)	0 (0–1)	0 (0–1)	0.60
CRP (mg/L)	5 (1–26)	4 (1–13)	5 (4–26)	< 0.001
DAS28CRP	1.8 (1.1–2.5)	1.7 (1.1–2.4)	1.9 (1.6–2.5)	0.002
HAQ (0–3)	0.125 (0–1.625)	0 (0–0.875)	0.31 (0–1.625)	0.002
VAS Global (mm) (0–100)	13 (0–67)	10 (0–45)	15 (0–67)	0.16
ACR-EULAR*	1.6 (0–7.7)	1.4 (0–5.3)	1.8 (0–7.7)	0.35
ACR-EULAR-remission*	77%	76%	79%	0.74
SDAI	2.1 (0.3–8.2)	1.6 (0.3–6.1)	2.8 (0.5–8.2)	0.05
SDAI-remission	75%	76%	74%	0.85
**Ultrasound assessment**				
Grey-scale sum score (0–72)	6 (0–19)	4 (1–18)	6.5 (0–19)	0.71
Doppler sum score (0–72)	0 (0–12)	0 (0–12)	0 (0–7)	1.00
Grey-scale sum score (MCP2–5 and wrist) (0–30)	3 (0–18)	3 (0–18)	3 (0–12)	0.25
Doppler sum score (MCP2–5 and wrist) (0–30)	0 (0–12)	0 (0–12)	0 (0–5)	0.84

Values are given as median (range) and percentage. Comparison between groups by Mann-Whitney’s test and Pearson chi-square with *p* < 0.05 considered significant

*DMARDs* disease-modifying anti-rheumatic drugs, *CRP* C-reactive protein, *HAQ* Health Assessment Questionnaire, *VAS* visual analogue scale

*Patients in ACR-EULAR Boolean-remission are identical to patients in CDAI-remission

Patients in the bDMARD group as compared to the csDMARD group had significantly longer disease duration; more patients had erosive disease, were anti-CCP positive and had higher CRP, HAQ, DAS28-CRP and SDAI-scores (Table 1). There were no differences in the fraction of patients fulfilling the SDAI-, CDAI- and ACR-EULAR Boolean-remission criteria in the two treatment groups.

Overall, very little clinical residual disease activity was present with 79 of the patients (91%) having neither clinically swollen nor tender joints in the 28 joints assessed for DAS28-CRP. Five patients (6%) had a maximum of 2 swollen joints and three patients (3%) had a maximum of 1 tender joint. None of these patients had both tender and swollen joints.

### Ultrasound findings

For the entire cohort, the GS sum score at patient level was median (range) 6 (0–19) and the Doppler sum score was 0 (0–12), with no significant differences between treatment groups. When evaluating ultrasound scores only from the hands, the pattern was similar with no significant difference between groups (Table 1).

When assessing 24 joints, 44% of the patients had Doppler score > 0 in at least one joint and 93% of the patients had a GS score > 1 in at least one joint while 47 patients (54%) had a GS score > 2 in at least one joint (no statistically significant difference according to treatment).

When assessing the hands only, 40% of the patients still had a Doppler score > 0, 93% of the patients had a GS score > 1 while 37% of the patients had a GS score > 2 in at least one joint (no statistically significant difference between treatments).

### Ultrasound remission

We only found strict ultrasound remission in 6 (7%) patients in the bDMARD group and in 0 (0%) patients in the csDMARD group (*p* = 0.01) (Table 2). In the entire cohort, 37% were in semi-strict ultrasound remission and 56% in Doppler ultrasound remission with no significant difference between treatment groups. Similar results were found for ultrasound remission among the subgroups of patients who, in addition to DAS28-CRP-remission, also fulfilled the ACR-EULAR Boolean-remission criteria, CDAI-remission criteria and SDAI-remission criteria (Table 2).

**Table 2:** Proportion of patients in ultrasound remission, according to different ultrasound remission definitions as well as different clinical remission criteria

**Patients in DAS28CRP-remission**				
	**All patients (*n*** **= 87)**	**csDMARD group (*n*** **= 45)**	**bDMARD group (*n*** **= 42)**	**Difference between treatment groups (*p*** **value)**
Ultrasound strict remission	6 (7%)	0 (0%)	6 (14%)	0.01
Ultrasound semi-strict remission	32 (37%)	15 (33%)	17 (40%)	0.50
Ultrasound Doppler remission	49 (56%)	26 (58%)	23 (55%)	0.78
Patients in ACR-EULAR Boolean-remission*				
	**All patients (*n*** **= 67)**	**csDMARD group (*n*** **= 34)**	**bDMARD group (*n*** **= 33)**	**Difference between treatment groups (*p*** **value)**
Ultrasound strict remission	6 (9%)	0 (0%)	6 (18%)	0.01
Ultrasound semi-strict remission	25 (37%)	12 (35%)	13 (39%)	0.73
Ultrasound Doppler remission	38 (57%)	21 (62%)	17 (49%)	0.40
**Patients in SDAI-remission**				
	**All patients (*n*** **= 65)**	**csDMARD patients (*n*** **= 34)**	**bDMARD patients (*n*** **= 31)**	**Difference between treatment groups (*p*** **value)**
Ultrasound strict remission	6 (9%)	0 (0%)	6 (19%)	0.007
Ultrasound semi-strict remission	26 (40%)	13 (38%)	13 (50%)	0.76
Ultrasound Doppler remission	38 (59%)	22 (58%)	16 (51%)	0.29

Values are given as number of patients and percentage. Comparison between groups by Pearson chi-square with *p* < 0.05 considered significant

*Ultrasound strict remission* no joints with GS score > 0 and Doppler score > 0, *ultrasound semi-strict remission* no joints with GS score > 1 and Doppler score > 0, *ultrasound Doppler remission* no joints with Doppler score > 0. *csDMARD* conventional synthetic disease-modifying anti-rheumatic drug, *bDMARD* biological disease-modifying anti-rheumatic drug, *GS* grey scale

*Patients in ACR-EULAR Boolean-remission are identical to patients in CDAI-remission

When assessing only the hands, numerically more patients were in semi-strict ultrasound and in Doppler ultrasound remission as compared to the more extensive 24-joint ultrasound assessment but with no difference between treatments (Table 3).

**Table 3:** Proportion of patients in ultrasound remission for the hands only according to different ultrasound remission cut-offs

Patients in DAS28CRP-remission				
	All patients (***n*** = 87)	csDMARD patients (***n*** = 45)	bDMARD patients (***n*** = 42)	Difference between treatment groups (***p*** value)
**Bilateral wrist and MCP2–5 only**				
Ultrasound strict remission	6 (7%)	0 (0%)	6 (14%)	0.009
Ultrasound semi-strict remission	43 (49%)	23 (51%)	20 (48%)	0.75
Ultrasound Doppler remission	52 (60%)	27 (60%)	25 (60%)	0.96

Values are given as number of patients and percentage. Comparison between groups by Pearson Chi-Square with *p* < 0.05 considered significant

*Ultrasound strict remission* no joints with GS score > 0 and Doppler score > 0, *ultrasound semi-strict remission* no joints with GS score > 1 and Doppler score > 0, *ultrasound Doppler remission* no joints with Doppler score, *csDMARD* conventional synthetic disease-modifying anti-rheumatic drug, *bDMARD* biological disease-modifying anti-rheumatic drug, *MCP* metacarpophalangeal joint, *GS* grey scale

## Discussion

The present study demonstrated subclinical synovitis by ultrasound in the majority of RA patients in longstanding clinical remission that had been obtained through a DAS28-CRP T2T-steered strategy in routine care. Furthermore, the patients had very little clinical residual disease activity with only five patients having a maximum of 2 swollen joints. Very few patients were found to be in strict ultrasound remission, and this was only observed in patients treated with bDMARD. Doppler ultrasound remission was obtained by 56% of patients and was more frequent than the strict and semi-strict ultrasound remission, and independent of treatment type. We had similar findings when assessing only the hands. As DAS28-CRP-remission state does not exclude minimal synovitis, we also assessed the number of patients fulfilling CDAI-, SDAI- and ACR-EULAR Boolean-remission criteria. The proportion of patients fulfilling the different ultrasound remission criteria in these groups were the same as for the DAS28-CRP-remission group.

Our findings of subclinical synovitis regardless of the clinical remission criteria applied and independent of the type of treatment are in line with other studies published over a period of more than 10 years [12–14, 16]. However, in our study, only 7% of the patients were in strict ultrasound remission and this was only observed in bDMARD-treated patients. This is overall less than observed in the study by Saleem et al. [13] where strict ultrasound remission was seen in 10% of the bDMARD-treated patients and in 16% of the csDMARD-treated patients. Other studies have reported even higher percentages in strict ultrasound remission (26–35%) for bDMARD-treated patients in clinical remission [14]. Such differences between studies most likely relate to differences in the applied scoring systems, hardware and differences in the ultrasonographer’s interpretation and scoring of Doppler findings. In the study by Saleem et al. [13], the Doppler activity was scored as none, mild, moderate and severe, and the ‘none’ and ‘mild’ scores were grouped as normal, which makes a direct comparison difficult, as any Doppler activity in our study would preclude a patient from being classified as in ultrasound remission.

As the optimal definition for ultrasound remission has yet to be determined, we explored different ultrasound remission criteria in the present study. Most emphasis is often put on the presence of Doppler activity independent of the GS score and we found 56% of the patients to fulfill the Doppler ultrasound remission criteria. Only 37% of the patients fulfilled the semi-strict remission criteria in which GS synovial hypertrophy score = 1 is accepted. The relevance of a GS score = 1 has been debated, especially in the feet where GS synovial hypertrophy is a frequent finding both in longstanding RA and in healthy controls [29, 30]. However, a GS score = 1 for synovial hypertrophy even without Doppler activity has been shown to change during treatment, also in the feet [31, 32]. Furthermore, GS synovial hypertrophy has previously been shown to be related to erosive progression over time [8]. It therefore appears justified to define true ultrasound remission as strict ultrasound remission with a GS score = 0 and a Doppler score = 0 despite that this appears difficult to achieve in routine care.

The patients in our study had obtained remission through a DAS28-CRP T2T-steered strategy as applied in routine care; however, the presence of subclinical synovitis appeared the same as in studies where the patients’ state of remission was determined by the treating physician and not obtained through a T2T strategy by the use of composite scores [6, 13]. DAS28-remission is often related to residual disease activity [4, 33, 34] whereas the ACR-EULAR Boolean-remission criteria better reflect true clinical remission with only minimal subclinical inflammation also by imaging [35–37]; however, no patients fulfilling the stricter ACR-EULAR Boolean-remission criteria were found to be in strict ultrasound remission in the csDMARD group, and only 7% of patients in the bDMARD-treated group. We found similar percentages of the patients in CDAI-, SDAI- and ACR-EULAR Boolean-remission to be in semi-strict ultrasound and in Doppler ultrasound remission as those in DAS28CRP-remission which is in line with a previous study [35].

Though recent studies have shown that ultrasound gives no added value in clinical practise for obtaining clinical remission in early RA patients [38, 39], the current study adds to the large amount of data where, when applying ultrasound for assessing remission, subclinical synovitis is found to be present in patients who has achieved clinical remission, including ACR-EULAR Boolean-remission, in routine care independent of clinical strategy and composite scores applied, number of joints assessed and ultrasound equipment used. Furthermore, data from previous studies have shown that erosive progression is related not only to the presence of Doppler activity in joints of RA patients in remission but also to the presence of GS synovial hypertrophy [8, 40] and that the presence of Doppler activity is related to the risk of flare and unsuccessful drug tapering [17–19, 40]. This consequently challenges the use of clinical composite scores alone for determining a state of remission in established RA patients in routine care. The current cross-sectional study cannot address whether patients in remission with subclinical synovitis should be treated differently than patients without subclinical synovitis and studies are needed in this area.

The strengths of our study are that data are representing a routine care setting where a T2T strategy was applied. We have used the OMERACT consensus-based and validated scoring system for scoring synovitis (GS and Doppler) and have assessed multiple joints providing a more comprehensive ultrasound status of the patients. The limitations are that the data are obtained from a single clinical centre and since it is cross-sectional, it does not contribute with additional data on erosive progression, flare or tapering. Further, the ultrasound-assessed joints are not completely identical to the joints involved in the DAS28. However, we found subclinical inflammation also in the clinically assessed joints.

## Conclusion

Subclinical synovitis detected by ultrasound is present in the vast majority of patients in longstanding DAS28-CRP-remission independent of the type of treatment when using a DAS28-CRP T2T-steered strategy in routine care. Remission based on clinical composite scores alone is very rarely equivalent to the complete absence of inflammation by ultrasound and may not be sufficient for establishing a state of remission. Further studies are needed to explore if patients with subclinical inflammation in contrast to patients without would benefit from different treatment strategies.

## Data Availability

The datasets used and/or analysed during the current study are available from the corresponding author on reasonable request.
